# The Safe Environment for Every Kid Model in the Swedish Child Health Services: Adoption and Introduction in a Healthcare Region

**DOI:** 10.1111/hex.70078

**Published:** 2024-10-23

**Authors:** Marie Golsäter, Ann‐Christine Andersson

**Affiliations:** ^1^ CHILD Research Group, School of Health and Welfare Jönköping University Jönköping Sweden; ^2^ The Child Health Care Service Region Jönköping County Jönköping Sweden; ^3^ Futurum—Academy for Health and Care Region Jönköping County Jönköping Sweden; ^4^ Jönköping Academy for Improvement of Health and Welfare, School of Health and Welfare Jönköping University Jönköping Sweden; ^5^ Department of Care Science Malmö University Malmö Sweden

**Keywords:** caregiver engagement, Child Health Services, collaboration, coordination, equity, integrated care, prevention, questionnaire, SEEK model

## Abstract

**Background:**

Early support for children and families in need can improve children's health and development. In a Swedish region, a new working model called Safe Environment for Every Kid (SEEK) was introduced in the Child Health Services to facilitate the early identification of psychosocial risk factors.

**Objective:**

The aim of this study was to describe the adoption and introduction of the SEEK model in the Child Health Services of Region Jönköping County.

**Methods:**

Quantitative data were analysed using descriptive statistics, whereas qualitative data were analysed separately by a thematic approach. The results were then interpreted together with documents (including CHS management meeting notes and documents describing planning and training sessions) using an exploratory mixed‐methods approach to give a comprehensive description of the adoption and introduction of the SEEK model.

**Results:**

The results show that the SEEK model improved coordination and collaboration, which led to better integrated care for children and families. The structure was regarded as supportive when introducing the SEEK model in Child Health Services. The questionnaire, as part of the SEEK model, was used in 88% of possible health visits. The most reported reason for not using it was a lack of time.

**Conclusions:**

The desire to make a difference and thereby promote better health and development for children was a crucial factor for the nurses in adopting the SEEK model in their clinical practice. The design using coaches was also appreciated and supported the adoption and introduction of the SEEK model.

**Patient or Public Contribution:**

Parents and healthcare professionals did not contribute to the research process. The results are based on dialogues between CHS nurses and parents after the parents filled in the SEEK questionnaire, providing an understanding of professional relationships when dealing with challenging issues.

**Abbreviations**
CHS: Child Health Services
PQ: Parent Questionnaire (within SEEK)
SEEK: Safe Environment for Every Kid

## 1 Introduction

In Sweden, the Child Health Services (CHS) is part of the primary care organization [1]. Based on national guidelines, the CHS offers regular health visits to promote children's health and development, as well as parenting support based on the individual child and family's needs. The CHS's services are voluntary, free of charge and include all children from birth to 6 years of age [1]. To promote children's health and development, the Safe Environment for Every Kid (SEEK) model [2] aimed to identify risk factors and provide further support to families with young children was adapted to Swedish conditions within the CHS and the social services in the framework of family centres [3].

There has been a growing interest in promoting children and families' well‐being through the early detection of risk factors [4]. Screening for risk factors in paediatric primary care improves the possibility of supporting the family needs [5]. To support families in an adequate way, the focus should be on young children, and the screenings should be repeated at several visits [4].

A study by Matson et al. [6] found that 87% of parents approve of clinics asking about family situations. At the same time, parents reporting concerns about substance use have lower trust in CHS personnel. Selvaraj et al. [5] found that 86% of parents screened for family risk factors were positive and wanted the screening to continue. Making the questioning routine for all families facilitates the acceptance of screenings in general [4, 7]. Professionals need repeated training and support to feel comfortable asking about risk factors and handling the outcomes, thereby promoting the health and well‐being of the child and family [4].

The CHS national guidelines [1] constitute a common, universal and equal programme for all children in Sweden. At the same time, CHS nurses must provide care based on individual needs. To improve person‐centredness and outcomes in healthcare, collaboration among different professions is highlighted as important [8]. In a review by Rawlinson et al. [9], the most common barriers to collaboration are a lack of long‐term efforts and leadership, a lack of time and adequate training, vague roles and responsibilities, doubts about the benefits and resistance to change. The SEEK model can be a tool helping CHS nurses to identify risk factors and, in collaboration with other professions, coordinate and provide further support to families with young children [2, 10]. In a pilot study, Swedish CHS nurses experienced that the SEEK model improved their opportunities to identify psychosocial risk factors and meet families' needs for psychosocial support, thereby promoting children's health and development [10].

## 2 SEEK Model

The SEEK model consists of four parts [3]:

- Education of healthcare personnel about psychosocial risk factors.
- The SEEK Parent Questionnaire (PQ), filled in by parents at routine child health visits.
- Dialogue between parents and CHS nurses based on the answers in the PQ and the family's needs and wishes.
- When needed, provision of further support or assistance for families using resources from healthcare or social services.

The PQ consists of 17 items grouped into six areas known to be risk factors: child safety, socioeconomic vulnerability, depression, parental stress, domestic violence and alcohol abuse. It is used in the CHS, with parents completing it at regular health visits when the child is 6–8 weeks, 10 months, 18 months, 2.5 years and 4 years old [3]. After the PQ is filled out, the CHS nurse and the parents have a dialogue about the answers and highlight issues that could affect the child and the family. Based on the needs and wishes of the parents, additional support at the CHS can be offered, or the family can be referred to a social worker at the family centre for additional support. A coordinated home visit by the CHS nurse together with a social worker from the family centre is another opportunity for additional support based on the outcome of the PQ.

### 2.1 Local Context

This article is part of a larger project following the introduction of the SEEK model in the CHS in Region Jönköping County in southeast Sweden. The region is one of Sweden's 21 autonomous bodies managing most of the healthcare for the population. The region has 13 municipalities and almost 370,000 inhabitants, of whom about 26,000 are children of preschool age [11]. In Region Jönköping County, there are 25 CHS units, all organized as family centres, with about 100 CHS nurses, social workers and other professionals offering integrated care. Each unit is led by a head of unit who reports to the Regional Head of the CHS. The CHS is led by a management team consisting of the Regional Head, a deputy department head, the five heads of unit, a development officer and an administrative coordinator.

### 2.2 Introducing New Working Models

Introducing new ways of working often involves difficulties, and different strategies have been used to overcome these [8, 12, 13]. The complexity in healthcare, including system properties and variation, interferes with putting new routines into action [14]. Greenhalgh et al. [15], studying the spread of innovations in health services, differentiate between passive diffusion, active and planned dissemination, active and planned mainstreaming implementation and sustainable routinization. Their review concludes that some important determinants involve communication, managers' attitudes and professional knowledge and engagement. People will also be more motivated to try out new working models if they experience there is a problem. Identifying common quality improvement goals is another motivation factor [16].

The strategy that is chosen for implementing an innovation is also crucial [15, 17, 18]. Common strategies involve staff education and printed information material. When introducing new routines, the adoption is more likely to succeed if issues involving the local context, such as organizational constraints, local practice and attitudes, are addressed beforehand [16, 19]. The ‘implementers’ need to understand how people respond to change [20], and Greenhalgh et al. [15] emphasize that the better planned and more active the process is, that is, more engagement, the greater the chances of success. Improvement tools are aimed at making healthcare better and safer by introducing the best ways of working. Therefore, improvement tools can be useful when introducing new ways of working [21].

### 2.3 The Project

**Figure 1:**
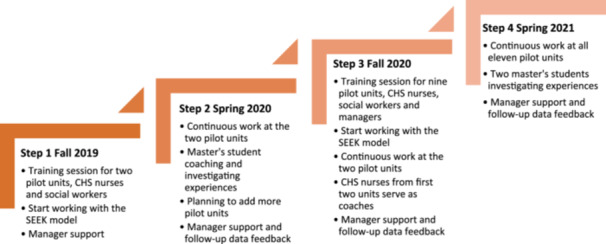
The four steps in the first phase of the introduction of the SEEK model.

The SEEK model was adopted and introduced in the region in several phases. This article describes the first phase (2019–2021) of the project, involving the adoption and small‐scale testing of the introduction in four steps (Figure 1). In the beginning, the staff was instructed to start with the families with younger children.

In the first step, the CHS nurses and social workers at two pilot units attended a 1‐day training session on the SEEK model in the fall of 2019. In the second step, a master's student coached the staff and followed the adoption and introduction at the two pilot units [22]. The project used improvement tools to conduct small‐scale tests [23] during steps 1 and 2. Another strategy was local training and support from coaches [24]. Improvement coaches trained in improvement methods and tools are frequently used in the region when introducing new ways of working. In the third step, nine additional child healthcare centres were included. The additional nine units were chosen by the CHS management aiming at an equal distribution in the region. The staff at these units attended a 1‐day training session. In this way, all CHS nurses and social workers involved were trained to use SEEK. At the training sessions, the CHS nurses received a booklet presenting the model and a manual on the local routines. To facilitate the decision on where to refer parent(s), a referral document was created, presenting the resources available for each municipality in the county. During the third and fourth steps, the CHS staff from the first two units acted as coaches to the new units added. Measurements and follow‐up data were used to give direct local feedback to the involved units. The experiences at the nine new units were investigated by two students undergoing their specialist training in paediatric nursing [25].

As shown in the literature [2, 3, 10], the SEEK model can be a useful tool to identify vital risk factors and provide early support to families. The region has started to introduce and use the SEEK model as a tool for improving the health and well‐being of children and families. It is important to explore how an introduction in a local context can be supported so that it is useful. A decision to introduce a new way of working seldom becomes successful without thoughtful support of the process [15]. Therefore, the aim of this article is to describe the first phase of adoption and introduction of the SEEK model in the CHS in Region Jönköping County.

## 3 Methods

### 3.1 Design

This article uses an exploratory mixed‐methods approach [26, 27]. The adoption and introduction of the SEEK model were explored through the collection of quantitative data, which then were compared, interpreted and described together with the qualitative results [26, 27, 28]. The material in this article consists of aggregated measurements from the regional CHS database during the same period as the qualitative interviews were conducted. Interview results used as secondary data from a 2‐year master's thesis project studying the two units in steps 1 and 2, and a 1‐year master's programme in paediatric nursing, studying the nine CHSs in steps 3 and 4, further described in the previous section [22, 25]. In addition, documents from the adoption and introduction phase were included.

### 3.2 Data Collection

The quantitative data in this study consist of the data in the action forms and PQs. After each health visit where the SEEK PQ was supposed to be used, the nurses filled in an action form containing information about the child's age (Table 1) and actions taken (Table 2). The action form also contained information on why the SEEK PQ was not used in some health visits. The action form, together with the PQ form data, was entered into the regional CHS database by the CHS personnel. The authors retrieved matrix data from the database at an aggregated anonymous level and transferred it to a statistics program (IBM SPSS 27).

**Table 1:** Distribution of the PQ in the different age groups.

Child's age	Percentage of the total number of PQs
6–8 weeks	24.9
10 months	21.4
18 months	20.2
2.5 years	19.0
4 years	13.8
No age specified	0.7
Total	100.0

**Table 2:** Actions taken after SEEK (more than one action was possible).

Action	Number of actions
Information/dialogue during visit	2183
New contact CHS nurse	99
Home visit CHS nurse	2
Home visit CHS nurse and SW	4
Referral to SW	261
Report of concern to social services	2
Referral to psychologist	41
Primary care centre	26
Referral to another agency	88
Total	2706

The secondary qualitative data consisted of results from earlier qualitative focus group interviews [29], conducted as part of the student projects. At the pilot units in steps 1 and 2, all CHS nurses and three out of four social workers were included in the interviews. From steps 3 and 4, additional CHS nurses were interviewed (Figure 1). In total, seven focus group interviews were performed, and the results of two student theses [22, 25] were used as secondary data in this study.

In addition, CHS management meeting notes and documents describing the planning and training sessions were included as the third data set.

### 3.3 Data Analysis

**Figure 2:**
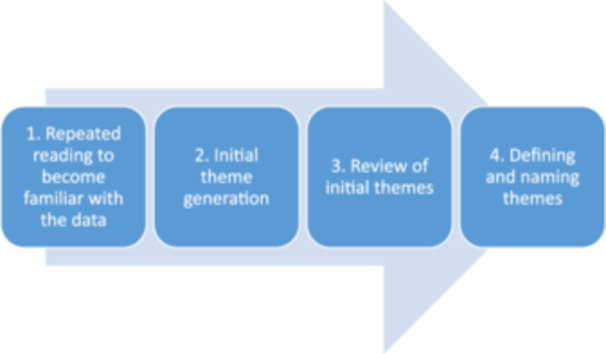
The thematic steps of analysis applied in this study, inspired by Braun and Clarke [30, 31].

Quantitative data were analysed using descriptive statistics in a statistics program (IBM SPSS 27). A qualitative synthesis inspired by a thematic approach [30, 31] was conducted (Figure 2). The results from two student theses were carefully read through by both authors, and initial themes were identified. The themes were sorted by similarity and thereafter reviewed and discussed until consensus was obtained, and thereafter named at a reflective level [31]. The reflexive approach was used to ensure transparency and acknowledge the potential influence of the researcher's perspectives on the analysis [31].

The management meeting notes and documents from planning and training sessions were analysed by abstracting information of importance in the process and then used in mixed‐methods interpretation. The authors read and identified key information, which responded to the aim. The results from the three data sets (the quantitative matrix data, the qualitative interview results and the meeting notes and documents) were then compared, interpreted and described together using an exploratory mixed‐methods approach [26, 27, 28].

## 4 Results

### 4.1 Quantitative Results

**Figure 3:**
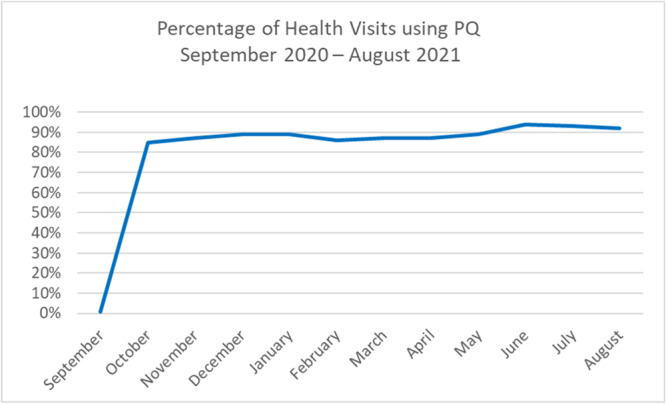
Use of Parent Questionnaire (PQ) during health visits per month.

From 1 September 2020 to 31 August 2021, the PQ was used at 6673 out of 7535 (88%) possible health visits. There was variation in the total number of possible health visits at which it was used, from 85% to 92%, depending on the month (Figure 3).

Looking at 11 participating units, the proportion of health visits at which the PQ was used varied between 83% and 98%, with a median value of 89%. The unit where the PQ was used most, where one of the units that was included already in step 1. When looking at the parents who answered the PQ, 78% were mothers, 20% were fathers, and the rest were unknown.

Regarding the reason for not using the PQ, the nurses stated that in 224 cases (26%), they forgot to take it out; in 413 cases (48%), a lack of time was cited as the cause; and in the other 225 cases (26%), another reason was given. Regarding other reasons, situations were mainly described as follows:

- The parents had already answered the PQ on a previous health visit a few weeks before the current one.
- The child was tired or sad.
- The family had a specific problem or need that became the focus of the visit.
- Linguistic difficulties/PQ was not available in the language that the parents spoke.
- The family arrived late to the visit.

Looking at the different age groups, the distribution of the PQ slightly decreases in relation to the child's increasing age (Table 1). This was also in line with the instruction to focus on the younger children to start with.

The distribution of outcomes on any issues in the PQ was 42% for all 6673, with variation between the 11 units from 29.9% to 49.1%. The distribution of outcomes in the different age groups showed variation from 38.9% (2.5 years) to 43.7% (10 months).

A total of 2706 actions were described, with information/dialogue during the health visit in 2183 cases and a return visit to the CHS nurse in 99 cases. In 261 cases the family was referred to a social worker at the family centre, and in a further 41 cases to a CHS psychologist. In an additional 88 cases, the family was referred to another professional for issues such as smoking cessation or family counselling (Table 2).

A total of 330 parents wanted to wait to deploy further support, and 665 parents indicated that they already had the support they needed.

### 4.2 Qualitative Synthesis

The synthesis of the two interview studies resulted in three themes: Challenges, Coordination and Collaboration, and Making a Difference.

In the *Challenges* theme, topics such as how to divide responsibilities were highlighted. Some CHS nurses experienced that a great deal of the responsibility was placed on them, but there was a difference between when the social workers actively took part in health visits and when they did not. When the social worker was more involved, it was easier to achieve shared responsibility and establish integrated care.

The CHS nurses accomplishing the visit and using the PQ needed support. The questions were not always easy to discuss, especially those involving finances, alcohol and violence, and there were some thoughts as to the honesty of the answers the parent(s) gave. Another challenge was the time aspect; sometimes the PQ took time from other duties, such as observing the child. The structure was sometimes experienced as unclear, but access to coaches could balance obstacles.

The *Coordination and Collaboration* theme entailed structures and the establishment of new mutually coordinated routines involving how CHS nurses and social workers can work together. At the same time, the CHS nurses believed that SEEK contributed to better collaboration. This can be noted in one of the most frequent actions being more referrals to other agencies, mainly to social workers at the family centre. This theme also increased the staff's understanding of the need to work together more closely around the families and increased their sense of security in working with the SEEK model.

The *Making a Difference* theme incorporated how personnel using the SEEK model got greater opportunities to identify and provide support that have a great impact on the families' situation. The PQ supported a deeper dialogue about difficult topics. The CHS nurses felt that using a questionnaire made it seem like someone else was responsible for these difficult questions. Using a tool with more sensitive questions (e.g., about abuse and drugs) universal to all parents made the issue easier to approach compared to just asking about it. It also helped them truly pay attention to the family's situation, which in turn helped to improve the child's situation. The structured PQ encouraged deeper dialogue with the parents/family, which in turn highlighted the whole family's situation. The CHS nurses thought that it reinforced and improved their work and contributed to making a difference for the children and families.

### 4.3 Document Compilation

The documents from planning and training and meeting notes imply that SEEK was introduced in a structured and strategic manner. The process entailed several steps, such as meetings with other regions using the model before the start, planning, training and assigning resources in the form of coaches. New routines consisted of instructions on how to use the SEEK PQ and what to document after the visit. The ten‐month visit was allocated a longer time slot, giving the CHS nurses more time to introduce the SEEK model to the parents. The staff were instructed to start using the SEEK PQ in families with younger children. Information about the SEEK model was also displayed on a digital board at the CHS centres, allowing parents to learn about the model in advance. The CHS managers were in contact with the social care managers, so all staff received the same coordinated training and information. There were also opportunities for CHS nurses and social workers to meet and discuss things with the intention of improving collaboration. The unit managers took an active part in discussions reflecting the CHS nurses' experiences and problem descriptions, and the management team frequently discussed the work at their meetings, including how to continue and develop it further, based on the unit's follow‐up data.

### 4.4 The Overall Interpretation of the Result

The three parts of the study were interpreted as a whole to offer a comprehensive description of the adoption and introduction of the SEEK model in Region Jönköping County. The first phase of introducing the SEEK model benefitted from the structure, as it was planned for and established in the organization. The managers, within both the CHS and social care, actively took part in meetings and discussions during the work and tried to offer support by providing resources and showing interest in the staff experiences. The coaches were one of the factors found to be important; another important factor was that continuous data follow‐ups were available at the unit level. Getting direct performance feedback from the CHS management improved motivation and engagement, and the experience of making a difference. This led to a rapid increase in using the SEEK PQ in health visits, starting with the younger children. However, a lack of time was described as a reason why the SEEK PQ was not used during a visit. There was also a fear of raising difficult issues with children present; the CHS nurses were therefore initially instructed not to include the oldest children, which is seen in the data on the use of the PQ in different age groups. The approach to implementing SEEK implied improved collaboration between the CHS nurses and social workers at the family centre.

## 5 Discussion

The purpose of this study was to describe the adoption and introduction of the SEEK model in Region Jönköping County. The most important statement concerned making a difference: the CHS nurses described that the SEEK PQ helped them discuss difficult issues with the families, which promoted the children's health and development at an early stage. This is in line with the goal in the national guidelines [1] and studies showing that helping families early improves children's health [4, 5, 10, 32]. The experiences that the SEEK model contributed to elaborating on and improving the work in supporting the families, in this way making a difference for the children, can be interpreted as having a great impact on the introduction of the SEEK model, in line with Greenhalgh et al. [15] Furthermore, the clear structure, including routines to follow, had a great impact on the success of the introduction. This was also found in the study by Engström, Randell, and Lucas [10], in which the nurses emphasized both the structured method and that SEEK was helpful in discussions with parents. At the same time, the nurses underscored the difficulty of raising hard subjects like alcohol use, which the CHS nurses in our study also expressed.

The most frequent action, besides information/conversations during the health visit, was referral to the social worker at the family centre. Even if the joint home visits did not increase, the CHS nurses experienced that the coordination and collaboration between the different caregivers improved (i.e., better integrated care [33, 34]). Working within the framework of a family centre, with the opportunity for close contact with a social worker, has also been described by CHS nurses as a facilitating factor when encountering mothers exposed to intimate partner violence [35]. The different competencies at the family centre, with easy access to the social worker for advice and possibilities for joint visits instead of simply making a referral, were highlighted by the CHS nurses in Anderzén Carlsson et al. [35], and these experiences could be used to further elaborate on the coordination and collaboration between CHS nurses and social workers when working with the SEEK model [9], aiming to address fragmentation of care and integrate the work further [36].

A model for interdisciplinary collaboration illuminates the social worker side of collaboration with healthcare [13]. As only three social workers were included in this study, their experiences were not extracted and analysed separately, their statements were included in the same data set as the CHS nurses, and therefore not interpreted differently in the interview material. However, the results correspond well with the suggested components of the collaboration model: Interdependence, Newly Created Activities, Flexibility, Collective Ownership, and Reflection on the Working Process [13]. A review examining the values of integrated care found some components important to facilitate multidisciplinary professionals when collaborating with other colleagues [34]. The most used components were collaboration and coordination, which also this study indicates.

To achieve integrated care, the persons involved need to participate on equal premises [36]. This is seen in our study as the SEEK model was a new working model at the family centres. It was important that neither the CHS nor the social workers own the task before. Instead, they received training together from the start. The interdisciplinary collaboration model describes influences that need to be considered in collaborative work, such as structure, professional roles, personal characteristics, and history of collaboration [13]. These were covered in this project; see Figure 1. Managers and leadership were deeply involved, and the SEEK model offers a clear structure, which the CHS nurses appreciated. The roles were defined, and a document stating where to refer families in need was developed in each municipality.

It is not always easy to introduce new ways of working [12, 20]. In line with the results of the present study, changes in healthcare organizations are more likely to be introduced successfully if it is easy to perceive the benefits for the target group and when professionals feel prepared [15, 37] and engaged [35]. Main barriers can include a lack of time and training, a lack of clear roles, fear of missing professional tasks and communication issues [9]. While most of these issues were not raised in this project, one that was mentioned involved time: having a dialogue about difficult questions must be allowed to take time, and lack of time was the most common reason to exclude the PQ during the visit. At the same time, the professionals experienced a benefit for the families using the SEEK model, which had an impact on the adoption of this new model, in line with Nilsen et al. [37]. However, some questions were described as challenging in the encounter with the parent(s); for example, those about violence. At the same time, in an earlier study, mothers considered it important that CHS nurses ask questions about factors that affect the child's health, such as drugs or violence in close relationships [5, 7]. The mothers highlighted the importance of everyone being asked the same questions as a routine so that no one feels stigmatized, which is in line with the purpose of the SEEK model. Asking all parents the same questions as in the SEEK model can be seen as one way to develop the CHS nurses' work to ensure children's health and development in an equal way. Dubowitz et al. [4] also suggest solutions to ease the use of screening instruments, such as making it universal rather than using it only for families at risk. However, it has to be taken into consideration that not all parents feel comfortable sharing their problems, and Matson [6] reports that parents with concerns about substance use have less trust in healthcare professionals.

First, some CHS nurses received training in the SEEK model, and they then served as coaches and facilitators for others. The coaches include ways to improve communication, co‐location and learning about each other's skills and roles [9]. This was very useful, according to the interview participants. Working like this, training and using coaches has proven to be a good way of working with change and improvements [24]. The unit that used the PQ most (98%) was the first pilot, with all staff receiving the training and then becoming coaches for other units. This shows that being part of the introduction and development improves the use of new ways of working [15, 16].

Working as a team was another aspect expressed in the interviews as being important. The history of collaboration was not experienced as a problem; in contrast, the region has been working with family centres for a long time, trying to facilitate and coordinate collaborative activities to integrate care. In a Swedish study, CHS nurses described working at family centres as facilitating teamwork and how the skills within the team improved when the different kinds of expertise were put together [8, 38].

The interview participants felt that the communication between the CHS nurses and the social workers had improved. A study by Widmark et al. [39] shows that this mutual respect and understanding is far from evident, but that establishing good working conditions, trust, and respect is crucial [9, 33, 40]. Much of this was related to organizational structures, which again demonstrates the importance of a well‐structured and planned project from the beginning [15], as was the case here.

Using improvement strategies, such as testing on a small scale and evaluating on an ongoing basis, has been found to ease the change process [16, 23]. Improvement tools were used in this project, starting up with a few CHS centres, learning from their process, and then widening the scope. In introducing working models already developed and tested elsewhere—like SEEK [3]—through the use of improvement methods, professionals have the opportunity to influence the work as it is introduced, which has been found to be important for success [37]. Drawing on the results of this study, we recommend that CHS or similar organizations structure the work and include and engage all concerned when initiating a new way of working. Using improvement methods could be a way to support the work, also including the need for management support and continuous feedback.

### 5.1 Limitations

This is the first report studying the introduction of the SEEK model in Region Jönköping County. Although the body of the material is small, it nonetheless indicates that the model is useful. The qualitative data emerged from student works and are used as secondary data in this study to interpret the quantitative data. First‐hand interviews could have offered a more detailed understanding and examples of the content and performance of the health visits. Furthermore, an observational study could contribute to a deeper understanding of how the SEEK model is used in the CHS context. The next step will be to introduce and start using the SEEK model at all family centres in the region. This will generate more data, and the evaluation will continue to gain more knowledge about this way of working in collaboration between the CHS and social worker professionals to integrate and improve care for children.

## 6 Conclusions

The most important finding of this study was the CHS nurses' desire to make a difference and promote better health and development for children. Coordination and collaboration increased as a result. The action that was used most often, in addition to dialogue during the visit, was further referral to the social worker. The planning and structure were other factors that were regarded as important and improved the preconditions for this way of working. The design using coaches was also appreciated and supported the adoption and introduction of the SEEK model. This approach to locally adopting and introducing new working methods seems to be useful and can therefore also benefit other healthcare contexts when implementing new ways of working.

## Author Contributions

M.G. and A.C.A. are responsible for the evaluation project and this study. M.G. was the main responsible party for collecting and analysing the quantitative data, and A.C.A. for the qualitative meta‐analysis. M.G. and A.C.A. then interpreted the results together. A.C.A. drafted the manuscript with support from MG, both authors approved the final version.

## Ethics Statement

The study, performed in accordance with the Declaration of Helsinki and Swedish research regulations, was approved by the Swedish Ethical Review Authority (reference number 2021‐04925). All participants consented to participate in the students' interview studies. Quantitative data were obtained at an aggregated level and is thus not possible to connect to a specific individual.

## Consent

Written information and consent to participate in the study were sought and obtained from all participants.

## Conflicts of Interest

The authors declare no conflicts of interest.

## Data Availability

All data generated or analysed during this study are included in this published article.
